# Protective Effect of Ciclopirox against Ovariectomy-Induced Bone Loss in Mice by Suppressing Osteoclast Formation and Function

**DOI:** 10.3390/ijms22158299

**Published:** 2021-08-02

**Authors:** Hye Jung Ihn, Jiwon Lim, Kiryeong Kim, Sang-Hyeon Nam, Soomin Lim, Su Jeong Lee, Jong-Sup Bae, Tae Hoon Kim, Jung-Eun Kim, Moon-Chang Baek, Yong Chul Bae, Eui Kyun Park

**Affiliations:** 1 Cell and Matrix Research Institute, Kyungpook National University, Daegu 41944, Korea; hjpihn@hanmail.net; 2 Department of Oral Pathology and Regenerative Medicine, School of Dentistry, Institute for Hard Tissue and Bio-tooth Regeneration (IHBR), Kyungpook National University, Daegu 41940, Korea; jiwonparadise@hanmail.net (J.L.); kileyong93@daum.net (K.K.); aay0805@naver.com (S.-H.N.); friendship1240@hanmail.net (S.L.); marhaul@hanmail.net (S.J.L.); 3 Research Institute of Pharmaceutical Sciences, College of Pharmacy, Kyungpook National University, Daegu 41566, Korea; baejs@knu.ac.kr; 4 Department of Food Science and Biotechnology, Daegu University, Gyeongsan 38453, Korea; skyey7@daegu.ac.kr; 5 Department of Molecular Medicine, School of Medicine, Kyungpook National University, Daegu 41944, Korea; kjeun@knu.ac.kr (J.-E.K.); mcbaek@knu.ac.kr (M.-C.B.); 6 Department of Oral Anatomy and Neurobiology, School of Dentistry, Kyungpook National University, Daegu 41940, Korea; ycbae@knu.ac.kr

**Keywords:** osteoclast, bone resorption, ciclopirox, osteoporosis

## Abstract

Postmenopausal osteoporosis is closely associated with excessive osteoclast formation and function, resulting in the loss of bone mass. Osteoclast-targeting agents have been developed to manage this disease. We examined the effects of ciclopirox on osteoclast differentiation and bone resorption in vitro and in vivo. Ciclopirox significantly inhibited osteoclast formation from primary murine bone marrow macrophages (BMMs) in response to receptor activator of nuclear factor kappa B ligand (RANKL), and the expression of genes associated with osteoclastogenesis and function was decreased. The formation of actin rings and resorption pits was suppressed by ciclopirox. Analysis of RANKL-mediated early signaling events in BMMs revealed that ciclopirox attenuates IκBα phosphorylation without affecting mitogen-activated protein kinase activation. Furthermore, the administration of ciclopirox suppressed osteoclast formation and bone loss in ovariectomy-induced osteoporosis in mice and reduced serum levels of osteocalcin and C-terminal telopeptide fragment of type I collagen C-terminus. These results indicate that ciclopirox exhibits antiosteoclastogenic activity both in vitro and in vivo and represents a new candidate compound for protection against osteoporosis and other osteoclast-related bone diseases.

## 1. Introduction

Bone homeostasis is controlled by coordinating the functions of bone cells, including osteoblasts and osteoclasts, in response to various stimuli. Osteoblasts are primarily responsible for the synthesis of the bone matrix, whereas osteoclasts are capable of degrading mineralized tissue by producing protons and proteolytic enzymes [1]. Disruption of the balance between the activities of these bone cells resulting from increased osteoclastic bone resorption can cause osteoporosis, periprosthetic osteolysis, and other bone diseases [2]. Osteoporosis is recognized as a serious health problem because of severe complications, including bone fractures. Currently, antiresorptive drugs are used, which improve bone strength; however, several adverse events have been reported [3,4], which indicates a need for alternative agents.

In general, multinucleated osteoclasts are generated by the fusion of mononuclear preosteoclasts. Macrophage colony-stimulating factor (M-CSF) and receptor activator of nuclear factor κB ligand (RANKL) are two cytokines that are required for osteoclastogenesis [1]. Upon binding to RANKL, receptor activator of nuclear factor κB (RANK) interacts with adaptor proteins, such as tumor necrosis factor (TNF)-receptor-associated factor 6 (TRAF6), to transduce signals to downstream targets [5,6]. RANKL–RANK signaling ultimately induces and activates nuclear factor of activated T cells cytoplasmic 1 (NFATc1). This is followed by the upregulation of osteoclast marker genes that contribute to osteoclast differentiation and function [7].

Drug repurposing is an effective and useful approach for drug discovery because it identifies alternative indications for approved drugs [8]. This strategy can save time, cost, and risks related to drug development compared with traditional drug discovery. Using an in vitro osteoclastogenesis assay for screening Food and Drug Administration (FDA)-approved drugs for antiosteoclastogenic activity, we observed a strong inhibitory effect of ciclopirox on RANKL-induced osteoclast differentiation. Ciclopirox and its olamine salt (ciclopirox olamine) are antifungal agents used primarily for the treatment of superficial fungal infections, and they exhibit similar pharmacological activities [9,10]. Ciclopirox also exhibits antibacterial effects against various Gram-negative and Gram-positive pathogenic bacteria through anti-inflammatory activity by inhibiting the arachidonic acid cascade, which leads to the inhibition of prostaglandin and leukotriene production [11,12]. In addition, ciclopirox was shown to exhibit antitumor effects in diverse cancer cell lines [13,14]. Zhou et al. reported that ciclopirox olamine suppresses tumor growth by inhibiting proliferation and promoting apoptosis of human breast carcinoma cells [14]. Besides infection, the accumulated evidence indicates that ciclopirox may possess therapeutic properties against other diseases including cancer, diabetes, and cardiovascular disease [15,16,17]. Animal research has revealed that the LD_50_ following oral, intravenous, and intraperitoneal administrations of ciclopirox olamine were 1700–3290, 71–79, and 83–172 mg/kg, respectively, in mice and rats [18]. In addition, topical ciclopirox olamine is considered to be well tolerated with a low incidence of adverse events observed in the clinical trials; thus, ciclopirox has a favorable toxicological profile, suggesting that it may be repositioned for the treatment of various human diseases [18].

In this study, we determined whether ciclopirox could be repurposed as an antiosteoclastogenic and/or antiresorptive agent for osteoclast-related bone diseases, such as osteoporosis. To verify the therapeutic potential of ciclopirox, we examined its effect on osteoclast differentiation and function and elucidated the molecular mechanism through which ciclopirox attenuates osteoclastogenesis and bone resorption. In addition, ovariectomy (OVX)-induced osteoporosis and estrogen (17β-estradiol; E2) replenishment were used to evaluate and compare the in vivo efficacy of ciclopirox.

## 2. Results

### 2.1. Effect of Ciclopirox on RANKL-Induced Osteoclast Differentiation

**Figure 1:**
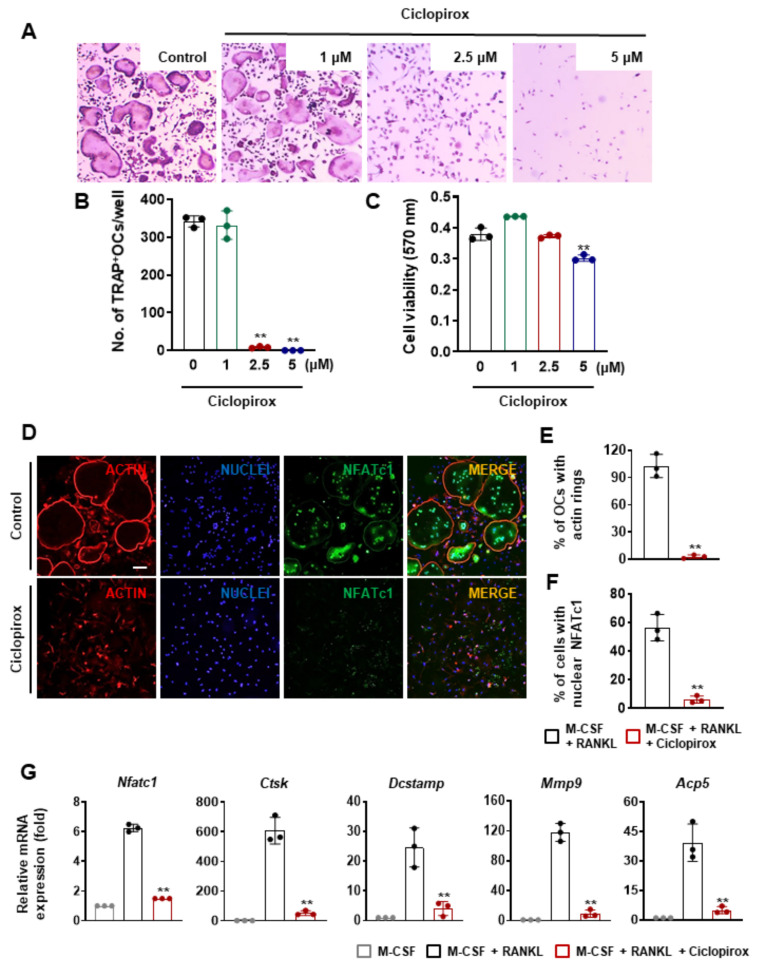
The effect of ciclopirox on receptor activator of nuclear factor kappa B ligand (RANKL)-mediated osteoclastogenesis. (**A**) Primary bone marrow macrophages (BMMs) were cultured in the presence of macrophage colony-stimulating factor (M-CSF) (10 ng/mL), RANKL (20 ng/mL), and various concentrations of ciclopirox for 4 days. The cells were stained for Acid Phosphatase, Leukocyte (TRAP). (**B**) The number of TRAP-positive multinucleated cells (≥3 nuclei) was calculated. (**C**) BMMs were incubated with M-CSF (10 ng/mL) and various concentrations of ciclopirox for 3 days. The methylthiazolyldiphenyl-tetrazolium bromide assay was performed to evaluate cell viability. (**D**–**G**) BMMs were cultured with M-CSF (10 ng/mL) and RANKL (20 ng/mL) in the presence or absence of 2.5 μM ciclopirox for 4 days. (**D**) The cells were fixed and probed with anti-nuclear factor of activated T cells cytoplasmic 1 (NFATc1) antibody. The nuclei and F-actin were then labeled with DAPI and rhodamine-conjugated phalloidin, respectively. Scale bar, 100 μm. Quantification of the percentage of (**E**) cells displaying actin rings and (**F**) cells expressing nuclear NFATc1. (**G**) The relative expression levels of osteoclast marker genes were measured by quantitative RT-PCR. Each experiment was performed three times independently. ** *p* < 0.01 (two-tailed Student’s *t*-test).

We initially screened 1163 FDA-approved drugs for their effects on osteoclast formation and cell viability using mouse primary osteoclast precursors. Bone marrow macrophages (BMMs) were cultured in an osteoclast induction medium with the drugs (1–5 μM) for 4 days. Of the drugs, ciclopirox exhibited a strong inhibitory effect on osteoclast differentiation. M-CSF and RANKL promoted the formation of tartrate-resistant acid phosphatase (TRAP)-positive multinucleated cells (MNCs); however, the addition of ciclopirox significantly reduced osteoclast formation from BMMs (Figure 1A). The number of TRAP-positive MNCs observed 4 days after treatment with 2.5 μM of ciclopirox was decreased by 97.5% compared with the vehicle-treated control, and the inhibitory effect of 2.5 μM of ciclopirox did not contribute to cytotoxicity (Figure 1B,C). Because an actin ring is a morphological characteristic of mature osteoclasts and crucial for resorption ability, we determined whether ciclopirox affects the formation of actin rings. F-actin rings were observed at the periphery of MNCs following RANKL stimulation for 4 days, whereas the formation of actin rings was significantly reduced in ciclopirox-treated (2.5 μM) cells (Figure 1D,E).

Since the transcription regulator, NFATc1, is important for osteoclast formation and activation, we evaluated its expression and localization to verify the suppressive effect of ciclopirox on osteoclast differentiation. Immunofluorescence staining revealed that most of the nuclei of the multinucleated osteoclasts were positive for NFATc1, which was expressed almost exclusively in the nucleus (Figure 1D). In contrast, the NFATc1 protein was hardly detectable in the nucleus when cells were treated with 2.5 μM of ciclopirox, showing an approximate 89% reduction compared with the control (Figure 1D,F). After 4 days of culture, the expression of Nfatc1 mRNA in 2.5 μM of ciclopirox-treated cells was also lower than that of the control (Figure 1G). As expected, treatment with ciclopirox resulted in downregulation of osteoclast marker gene expression, including cathepsin K (*Ctsk*), dendritic cell-specific transmembrane protein (*Dcstamp*), matrix metalloproteinase 9 (*Mmp9*), and TRAP (*Acp5*), which was consistent with reduced osteoclast formation (Figure 1G).

### 2.2. Effect of Ciclopirox on Osteoclast Function

**Figure 2:**
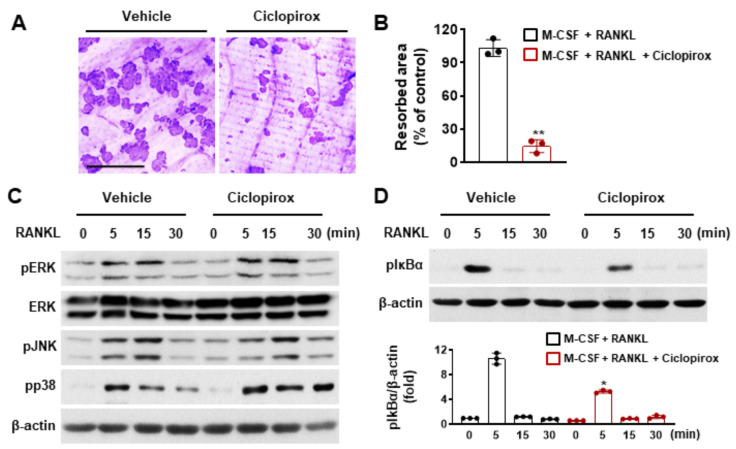
The effect of ciclopirox on osteoclastic bone resorption and receptor activator of nuclear factor kappa B ligand (RANKL)-induced signaling in bone marrow macrophages (BMMs). (**A**,**B**) BMMs were cultured on bone slices with macrophage colony-stimulating factor (M-CSF) (10 ng/mL) and RANKL (20 ng/mL) for 3 days and then treated with ciclopirox (2.5 μM) or vehicle. Resorbed areas visualized by hematoxylin staining were measured. Scale bar, 250 μm. (**C**,**D**) Serum-starved BMMs were treated with ciclopirox (2.5 μM) or vehicle for 1 h and then stimulated with RANKL (50 ng/mL) for various times. Immunoblotting was done using phosphospecific antibodies, and total ERK and β-actin were used as the loading controls. Each experiment was independently performed three times, except for that in C; data for the experiment shown in C were derived from two independent experiments. * *p* < 0.05, ** *p* < 0.01 (two-tailed Student’s *t*-test).

Since mature osteoclasts are primarily responsible for the degradation of mineralized tissues, the effect of ciclopirox on the bone resorption activity of osteoclasts was investigated using a bone resorption pit assay. Numerous resorption pits on bone slices were observed in the vehicle-treated control, whereas 2.5 μM of ciclopirox treatment suppressed pit formation (Figure 2A). The bone surface occupied by the pits was significantly decreased by 83.4% compared with the control (Figure 2B), indicating that ciclopirox directly affects osteoclast function.

### 2.3. Effect of Ciclopirox on RANKL-Mediated Signaling Pathways

As signaling through RANKL leads to the induction of NFATc1 and is crucial for osteoclastogenesis and bone-resorbing function, the effect of ciclopirox on RANKL-mediated signaling pathways was examined to understand the mechanism through which ciclopirox suppresses osteoclast formation and function. RANKL exposure induced phosphorylation of several MAPKs (p38, ERK, and JNK) and IκBα. In contrast, pretreatment with 2.5 μM ciclopirox suppressed RANKL-mediated phosphorylation of IκBα but not MAPK activation (Figure 2C,D).

### 2.4. Effect of Ciclopirox on OVX-Induced Bone Loss

**Figure 3:**
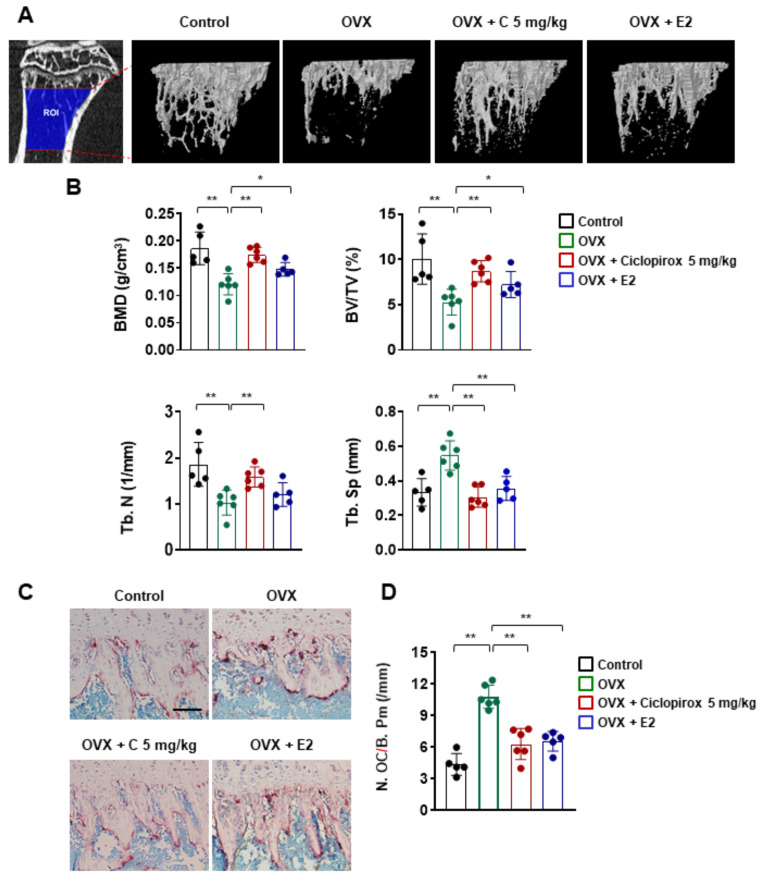
The effect of ciclopirox on ovariectomy (OVX)-induced bone loss in vivo. (**A**) Representative image of the trabecular region of interest (ROI: 0.21–1.94 mm from the growth plate) and three-dimensional images of the tibial trabecular bone were obtained. (**B**) Morphometric analysis of the trabecular bone in each group. Bone mineral density (BMD), bone volume fraction (BV/TV), trabecular number (Tb. N), and trabecular separation (Tb. Sp) were measured. Control *n* = 5; OVX *n* = 6; OVX + C 5 mg/kg *n* = 6; OVX + E2 *n* = 5. (**C**) Representative images of Acid Phosphatase, Leukocyte (TRAP)-stained tibial sections. (**D**) Quantitation of TRAP-positive cells in the proximal tibia. Scale bar, 100 μm. * *p* < 0.05, ** *p* < 0.01 (analysis of variance with Tukey’s post hoc). E2: 17β-estradiol, C: ciclopirox.

To further evaluate the in vivo efficacy of ciclopirox on bone loss, we established an OVX-induced osteoporosis mouse model. Three-dimensional images of the tibia’s trabecular bone showed that mice that underwent bilateral ovariectomy exhibited decreased trabecular bone mass (Figure 3A). Microstructural parameters, including bone mineral density (BMD), bone volume fraction (BV/TV), and trabecular number (Tb. N.), were significantly lower compared with those in the sham control group (Figure 3B). However, the values for these bone morphometric parameters were higher in both ciclopirox- and E2 (positive control)-treated ovariectomized groups compared with the OVX group, indicating that ciclopirox prevented OVX-induced trabecular bone loss (Figure 3B). Furthermore, TRAP staining revealed that ciclopirox treatment suppressed the formation of TRAP-positive osteoclasts and the number of osteoclasts in the ciclopirox-treated group was approximately 38% lower than that of the OVX group (Figure 3C,D).

### 2.5. Effect of Ciclopirox on Serum Levels of Bone Turnover Markers

**Figure 4:**
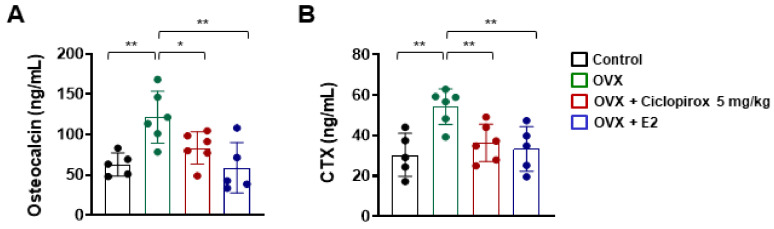
The effect of ciclopirox on biochemical markers of bone turnover. The serum levels of (**A**) osteocalcin and (**B**) CTX were quantified using ELISA kits. Control *n* = 5; OVX *n* = 6; OVX + C 5 mg/kg *n* = 6; OVX + E2 *n* = 5. * *p* < 0.05, ** *p* < 0.01 (analysis of variance with Tukey’s post hoc). E2: 17β-estradiol.

We determined the effects of ciclopirox on biochemical markers of bone formation (osteocalcin) and bone resorption (CTX). Compared with control mice, serum concentrations of osteocalcin and CTX were significantly increased in the OVX group (Figure 4). However, ciclopirox treatment ameliorated elevated serum levels of these bone turnover markers compared with the OVX group (Figure 4).

## 3. Discussion

Osteoclast-targeting agents including bisphosphonates (BPs) and denosumab are effective at preserving and improving bone mass and are commonly used medications for treating patients with osteoporosis. However, serious complications, such as antiresorptive drug-related osteonecrosis of the jaw, have been reported [19,20]. Atypical fracture associated with prolonged BP therapy and severe hypocalcemia in renal patients treated with denosumab have also emerged as complicating issues [3,4]. In this study, we screened a panel of FDA-approved drugs to identify compounds capable of suppressing osteoclast differentiation. To our knowledge, this is the first study to demonstrate that ciclopirox has significant potential as an alternative drug for managing excessive-osteoclast-activity-related bone disease. Ciclopirox significantly inhibited not only osteoclastogenesis through the downregulation of NFATc1 and its target genes but also bone resorption in vitro and prevented OVX-induced osteoporosis in vivo. 

The mechanisms through which ciclopirox regulates RANKL-induced osteoclast differentiation were elucidated by measuring the expression of osteoclast marker genes. Ciclopirox treatment led to a reduction in the expression of NFATc1 and its target genes that are associated with osteoclastogenesis (Figure 1G). Of these, DC-STAMP is a key molecule for cell fusion of mononuclear osteoclasts and multinucleation is an important process in osteoclastic bone resorption [21,22]. Although mononuclear osteoclasts derived from DC-STAMP-deficient mice resorb bone, they exhibit reduced bone-resorbing activity [22]. Wang et al. reported the critical role of actin filaments in preosteoclast migration and cell fusion [23], and the rearrangement of the actin cytoskeleton is required for bone resorption [24]. F-actin and TRAP staining revealed that ciclopirox treatment suppressed multinucleation and actin ring formation (Figure 1). Additionally, the resorbed area on the bone slice was decreased by ciclopirox (Figure 2A), indicating that ciclopirox exhibits antiresorptive activity. NFATc1 is an essential regulator of osteoclast differentiation and activation, and the NF-κB signaling pathway is important for the initial induction of NFATc1 [25,26]. The p50 and p65 NF-κB components bind transiently to the *NFATc1* promoter in response to RANKL stimulation, and the pharmacological inhibition of NF-κB results in a decreased NFATc1 expression and reduced osteoclast formation [25,27]. In addition, ablation of the p50 and p52 NF-κB subunits results in osteopetrosis in mice because of a defect in osteoclastogenesis [28]. This indicates that NF-κB signaling is important for the regulation of osteoclast differentiation by modulating NFATc1 expression. We found that pretreatment with ciclopirox reduced RANKL-induced phosphorylation of IκBα (Figure 2D). This suggests that its suppressive effect on osteoclast formation is mediated through downregulation of the NF-κB/NFATc1 signaling pathway, which is reflected by reduced levels of nuclear NFATc1 (Figure 1D). Although ciclopirox sustained the phosphorylation of p38, the integrity of the signaling cascades is critical for osteoclastogenesis.

Having established antiosteoclastogenic and antiresorptive activities in vitro, we investigated the in vivo therapeutic efficacy of ciclopirox on OVX-induced bone loss. Osteoporosis belongs to excessive-osteoclast-activity-related bone diseases, and there is an OVX animal model that mimics menopause-induced bone loss. Estrogen deficiency after menopause leads to increased osteoclastic bone resorption and risk of fracture [29,30]. Consistent with previous studies [31,32,33], mice that underwent bilateral ovariectomy exhibited a significant reduction in bone parameters (Figure 3B) and uterus index (data not shown) compared with mice in the sham-operated control group. The structural parameters of the trabecular bone obtained from micro-CT analysis revealed that ciclopirox prevents trabecular bone loss following ovariectomy to a similar extent as E2 (Figure 3B). The number of osteoclasts revealed by TRAP staining was decreased in ciclopirox-treated OVX mice compared with vehicle-treated OVX mice (Figure 3C). This indicates that the protective effect of ciclopirox against bone loss may be associated with a significant suppression of osteoclast formation. The maintenance of skeletal homeostasis relies on the balance between bone formation and resorption [34]. Although ciclopirox inhibited osteoclast differentiation and bone resorption in vitro and prevented bone loss in an experimental model in vivo, its effects on osteoblast differentiation remain to be elucidated. 

In conclusion, our results demonstrate that ciclopirox suppresses the expression of NFATc1 and its target genes and attenuates IκBα phosphorylation in response to RANKL. Ciclopirox also exhibited protective effects against bone destruction by inhibiting osteoclast formation, thus, providing novel insights into the treatment and management of osteoclast-related bone diseases.

## 4. Materials and Methods

### 4.1. Reagents and Antibodies

Ciclopirox was purchased from Selleckchem (Houston, TX, USA). Fetal bovine serum and α-minimal essential medium (α-MEM) were purchased from Gibco BRL (Grand Island, NY, USA). Recombinant murine M-CSF and RANKL were obtained from R&D Systems (Minneapolis, MN, USA). The Acid Phosphatase, Leukocyte (TRAP) staining kit, methylthiazolyldiphenyl-tetrazolium bromide (MTT), and dimethyl sulfoxide (DMSO) were purchased from Sigma-Aldrich (St. Louis, MO, USA). Antibodies against phospho-p38, phospho-ERK, ERK, phospho-JNK, and phospho-IκBα were purchased from Cell Signaling Technology (Danvers, MA, USA). Antibodies against NFATc1 and β-actin were purchased from Santa Cruz Biotechnology (Santa Cruz, CA, USA) and Sigma-Aldrich (St. Louis, MO, USA), respectively. Alexa Fluor 488 conjugated anti-mouse IgG secondary antibody was purchased from Thermo Fisher Scientific Inc. (Rockford, IL, USA). 

### 4.2. Animals

Male 5-week-old and female 8-week-old C57BL/6 mice were obtained from Dae Han Bio Link (Chungbuk, Korea) and allowed to acclimatize for 1 week. All animal experiments were approved by the Institutional Review Board of Kyungpook National University on the care and use of animals for research and were conducted in accordance with established guidelines for the care and use of laboratory animals.

### 4.3. Osteoclast Differentiation

Murine bone marrow cells were obtained from the femur and tibia of 6-week-old male C57BL/6 mice and incubated overnight in α-MEM containing 10% FBS [35,36]. Nonadherent cells were collected and cultured in the presence of M-CSF (30 ng/mL) for 3 days. The adherent cells were used as bone marrow macrophages (BMMs). For osteoclast differentiation, BMMs (10^4^ cells/well) were seeded into 96-well plates and cultured with M-CSF (10 ng/mL) and RANKL (20 ng/mL) in the presence or absence of ciclopirox. At the end of the incubation, the cells were fixed and stained using the Acid Phosphatase, Leukocyte (TRAP) staining kit for the quantification of TRAP-positive multinucleated cells (MNCs) containing three or more nuclei.

### 4.4. Cell Viability Assay

BMMs were incubated with M-CSF (10 ng/mL) in the presence or absence of the indicated concentrations of ciclopirox. After 3 days of treatment, the MTT colorimetric assay was used to assess cytotoxicity [37]. The cells were incubated with MTT for 2 h, the insoluble formazan crystals were dissolved in DMSO, and the absorbance of the wells at 570 nm was measured using a 96-well microplate reader (Bio-Rad Laboratories, Hercules, CA, USA).

### 4.5. Quantitative Real-Time PCR

The expression of TRAP (*Acp5*), cathepsin K (*Ctsk*), dendritic cell-specific transmembrane protein (*Dcstamp*), matrix metalloproteinase 9 (*Mmp9*), and nuclear factor of activated T cells cytoplasmic 1 (*Nfatc1*) mRNA were quantified by real-time (RT) PCR, as previously described [38]. Complementary DNA was synthesized from 1 μg of isolated RNA using SuperScript II Reverse Transcriptase (Invitrogen, Carlsbad, CA, USA). Quantitative RT-PCR was carried out using a LightCycler 1.5 real-time PCR system (Roche Diagnostics, Basel, Switzerland) and SYBR Premix Ex Taq (Takara Bio Inc., Shiga, Japan). The quantitative analysis of gene expression was performed by the 2^−ΔΔCt^ method; the sequence of primers used was as follows: *Acp5* (63 bp), 5′-TCCCCAATGCCCCATTC-3′ and 5′-CGGTTCTGGCGATCTCTTTG-3′; *Ctsk* (66 bp), 5′-GGCTGTGGAGGCGGCTAT-3′ and 5′-AGAGTCAATGCCTCCGTTCTG-3′; *Mmp9* (77 bp), 5′-AAAGACCTGAAAACCTCCAACCT-3′ and 5′-GCCCGGGTGTAACCATAGC-3′; *Dcstamp* (64 bp), 5′-CTTCCGTGGGCCAGAAGTT-3′ and 5′-AGGCCAGTGCTGACTAGGATGA-3′; *Nfatc1* (72 bp), 5′-ACCACCTTTCCGCAACCA-3′ and 5′-TTCCGTTTCCCGTTGCA-3′.

### 4.6. Immunoblotting

Total protein was isolated using a lysis buffer containing protease (iNtRON Biotechnology, Seongnam, Korea) and phosphatase inhibitors (GenDEPOT, Barker, TX, USA). Equal amounts of protein (25 μg) were separated on 10% sodium dodecyl sulfate-polyacrylamide gels and transferred to nitrocellulose membranes (Whatman, Florham Park, NJ, USA). The membranes were blocked with 3% nonfat dry milk in Tris-buffered saline containing 0.1% Tween 20, incubated with primary antibodies (1:1000) at 4 °C, followed by incubation with horseradish-peroxidase-conjugated secondary antibodies (1:2000). The membranes were developed using an enhanced chemiluminescent (Advansta, Menlo Park, CA, USA) reagent. The intensity of each protein band was measured using ImageJ.

### 4.7. Bone Resorption Assay

The pit formation assay was performed using bone slices (IDS Nordic, Herlev, Denmark) as previously described [38,39]. In brief, BMMs plated on bone slices were cultured with M-CSF (10 ng/mL) and RANKL (20 ng/mL) for 3 days. The cells were then treated with ciclopirox (2.5 μM) or vehicle for 24 h. After removing the cells, the bone slices were immersed in hematoxylin and the resorbed pit area was quantified using the i-Solution image analysis program (IMT i-Solution; Daejeon, Korea).

### 4.8. Immunofluorescence Staining

BMMs were seeded onto glass coverslips and cultured for 4 days in the presence of M-CSF (10 ng/mL) and RANKL (20 ng/mL) with or without ciclopirox (2.5 μM). The cells were then fixed and probed with an anti-NFATc1 monoclonal antibody followed by an Alexa Fluor 488 conjugated secondary antibody. After washing, the cell nuclei and F-actin were stained with 4′,6-diamidino-2-phenylindole dihydrochloride (DAPI; Santa Cruz Biotechnology, Santa Cruz, CA, USA) and rhodamine-conjugated phalloidin (Cytoskeleton, Denver, CO, USA), respectively. The cells were observed under a Leica DM 2500 fluorescence microscope (Leica Microsystems, Wetzlar, Germany).

### 4.9. Ovariectomy Animal Model

After 1 week of acclimatization, 9-week-old C57BL/6 female mice were randomly divided into four groups: sham-operated (Control, *n* = 5), surgically ovariectomized (OVX, *n* = 6), surgically ovariectomized with ciclopirox (OVX + C 5 mg/kg, *n* = 6), and surgically ovariectomized with 17β-estradiol (E2) (OVX + E2, *n* = 5) as described previously [31]. A week after surgery, the mice were administered vehicle, 5 mg/kg body weight of ciclopirox, or 10 μg/kg body weight of E2 by intraperitoneal injection six times per week. The treatment dose of ciclopirox was determined based on previously reported animal studies [18,40], and no adverse events were observed during the experiment. After 4 weeks, all mice were sacrificed by cervical dislocation and the long bones and blood were collected. The long bones were fixed for histological and microcomputed tomography (micro-CT) analysis, and blood samples were prepared for serum biochemical analysis.

### 4.10. Micro-CT and Histomorphometric Analysis

The fixed long bones were analyzed using a SkyScan 1272 high-resolution micro-CT system (Bruker, Kontich, Belgium). The scanning parameters were set at 60 kV/166 µA with a resolution of 8 µm for long bones. Three-dimensional images of the tibial trabecular bones (ROI: 0.21–1.94 mm from the growth plate) were obtained using CTAn/CTvol software (Bruker, Kontich, Belgium), and morphometric parameters including bone volume fraction (BV/TV), bone mineral density (BMD), trabecular separation (Tb. Sp.), and trabecular number (Tb. N.) were calculated using CTAn software (Bruker; Kontich, Belgium) as reported previously [31,35]. For histological examination, the fixed long bones were decalcified in 10% EDTA, embedded in paraffin, and serial sections were cut to a thickness of 6 μm. The sections were stained with TRAP and used to quantify osteoclast number per bone parameter.

### 4.11. Measurements of Bone Turnover Markers

Serum levels of osteocalcin (bone formation) and CTX (bone resorption) were determined using the Mouse Osteocalcin EIA kit (Biomedical Technologies, Stoughton, MA, USA) and the RatLaps (CTX-I) ELISA kit (Nordic Bioscience Diagnostics, Herlev, Denmark), respectively.

### 4.12. Statistical Analysis

All experiments were conducted separately at least two or three times, and the results are presented as the mean ± standard deviation. The statistical significance was assessed using a two-tailed Student’s *t*-test or one-way ANOVA with Tukey’s multiple comparison post hoc test. A value of *p* < 0.05 or *p* < 0.01 was considered statistically significant.

## Data Availability

The data presented in this study are available upon reasonable request from the corresponding author.
